# Bridging interoception and time perspective: toward an embodied model of consciousness

**DOI:** 10.3389/fpsyg.2026.1725236

**Published:** 2026-04-02

**Authors:** Olga Klamut, Simon Weissenberger

**Affiliations:** 1 Department of Psychiatry, First Faculty of Medicine, Charles University, Prague, Czechia; 2 Department of Psychology, Anglo-American University, Prague, Czechia

**Keywords:** interoceptive awareness, time perspective, temporal cognition, embodied consciousness, autonomic regulation, somatic functioning, neurovisceral integration, self-regulation

## Abstract

**Background:**

Emerging evidence suggests that the ability to sense internal bodily signals, interoceptive awareness, is central to embodied consciousness and adaptive self-regulation. Yet, the cognitive mechanisms by which interoceptive processes shape the continuity of conscious experience remain insufficiently understood. One such mechanism may be time perspective, a framework reflecting individuals’ orientation toward the past, present, and future, which structures how consciousness unfolds in temporal context.

**Methods:**

In a non-clinical sample of 152 adults, participants completed validated measures of interoceptive awareness (MAIA), time perspective (ZTPI), and self-rated indicators of somatic experience, including sleep quality and digestion.

**Results:**

Analyses showed that individuals with higher interoceptive awareness reported more adaptive somatic functioning, and that a balanced time perspective partially accounted for these associations. This suggests that interoception influences conscious self-regulation not only through autonomic processes, but also via temporal cognition.

**Conclusion:**

These findings support a neurocognitive framework of embodied consciousness, in which interoceptive awareness and temporal orientation interact to maintain psychological and physiological stability. By linking bodily awareness with temporal cognition, this study provides preliminary empirical evidence for a functional feedback loop that grounds conscious experience in the body and time. This perspective opens avenues for future experimental and longitudinal research, as well as for clinical applications such as mindfulness and time-oriented interventions to strengthen embodied self-awareness and temporal balance.

## Introduction

The ability to consciously attune to and regulate internal states is fundamental to maintaining both physiological and psychological health. Two constructs central to this capacity are interoceptive awareness - the perception of internal bodily signals; and time perspective - the cognitive-affective orientation toward the past, present, and future. While traditionally examined in separate literatures, recent interdisciplinary work suggests that these domains play converging roles in shaping conscious self-regulation and somatic functioning.

Interoception refers to the conscious perception and appraisal of internal bodily cues, including respiration, heartbeat, hunger, and visceral sensations (Mehling et al., 2018; Bornemann et al., 2014). It contributes to emotion regulation, behavioral flexibility, and adaptive stress recovery, and is increasingly recognized as a key determinant of physical and mental well-being. Disruptions in interoception are common across conditions involving altered states of bodily awareness, such as anxiety, trauma, and psychosomatic presentations (Pang et al., 2019; Schmitz et al., 2023; Smith et al., 2021).

Time perspective, conceptualized by Zimbardo and Boyd (1999), represents individuals’ orientation toward time across multiple frames: Past Negative, Past Positive, Present Hedonistic, Present Fatalistic, and Future. A balanced time perspective, characterized by high Future and Past Positive and low Past Negative and Present Fatalistic orientations; has been linked to adaptive stress regulation, life satisfaction, and psychological flexibility (Stolarski et al., 2011; Stolarski et al., 2015). In contrast, rigid temporal biases, such as excessive past-negativity or future disengagement, predict emotional dysregulation and maladaptive coping (Papastamatelou et al., 2015; Rönnlund and Carelli, 2018).

The ZTPI and MAIA emerge from distinct research traditions, yet both capture trait-like components of conscious self-regulation. The ZTPI quantifies deviation from balanced temporal orientation, which has been linked to emotional and autonomic dysregulation. The MAIA assesses multiple dimensions of interoceptive awareness - including self-regulation, trusting, and attention regulation - associated with emotion regulation, well-being, and parasympathetic tone (Mehling et al., 2018; Rönnlund et al., 2018).

Neurophysiological models offer a bridge between these domains. The anterior insula, central to interoceptive signal integration, is also involved in subjective time perception (Craig, 2009; Di Lernia et al., 2018; Wittmann and Meissner, 2022). The neurovisceral integration model (Thayer and Lane, 2000; Thayer and Koenig, 2025; Smith et al., 2017) provides a framework for understanding how flexible autonomic regulation - mediated by prefrontal, cingulate, and insular networks - supports adaptive conscious regulation. Similarly, polyvagal theory emphasizes autonomic pathways in stress and social engagement (Porges, 2011).

Other perspectives further highlight the systemic integration of bodily and psychological processes. Core autonomic functions such as sleep and digestion are highly sensitive to stress, and their disruption is common in trauma, anxiety, and chronic inflammatory conditions (Chrousos and Gold, 1992; De Bergeyck and Geoffroy, 2023; Walker and Druss, 2016; Harvey, 2002; Irwin, 2015). Early adversity, trauma, and chronic stress influence both interoceptive capacity and temporal bias (Meaney, 2010; Danese and Lewis, 2017), shaping how individuals inhabit their embodied present. Psychoneuroimmunological models extend this view by showing how inflammation and neuroendocrine dysregulation alter long-term cognitive–emotional functioning (Ader et al., 1995; Bower and Kuhlman, 2023; Buric, 2025; Slavich and Cole, 2013). Taken together, these findings support the idea that both interoception and time perspective reflect embodied imprints of lived experience, situated at the intersection of neural, autonomic, and immune processes.

Although time perspective refers to stable, trait-level orientations toward the past, present, and future, research in experimental psychology has long examined how humans perceive short intervals of time. Classical pacemaker–accumulator or “mental clock” models propose that perceived duration depends on an internal timing mechanism whose rate is influenced by arousal and whose output is shaped by attention (Gibbon et al., 1984; Meck, 1996; Meck and Benson, 2002). In these models, heightened arousal tends to accelerate the internal clock, leading to duration overestimation, while attention determines how much temporal information is processed. Importantly, attention here refers to the allocation of cognitive resources and should not be equated with conscious awareness itself.

While such models effectively explain interval timing, they do not fully account for how temporal processing becomes integrated into the ongoing experience of a continuous present or into broader temporal orientations such as time perspective. Recent accounts suggest that interoceptive signaling - continuously mediated by insular and cingulate networks - provides a dynamic representation of internal bodily states that unfolds in real time (Craig, 2009; Wittmann, 2014). From this perspective, attention may regulate access to both temporal and bodily signals, whereas interoceptive integration contributes to the felt continuity of experience. Interoception therefore does not replace attentional timing mechanisms; rather, it may complement them by linking moment-to-moment temporal processing with the embodied organization of conscious experience.

In this context, we introduce the concept of embodied time perspective to capture both neurocognitive and experiential dimensions of conscious experience. On one hand, the insula links interoceptive processing with subjective time perception, suggesting a shared neural basis (Craig, 2009; Wittmann, 2014). On the other hand, bodily awareness is accessed in the present moment, as sensory systems operate in real time. Experiences of past and future are therefore continually filtered through this embodied present, which serves as the immediate ground of consciousness.

A balanced time perspective is associated with psychological resilience and stress regulation (Zimbardo and Boyd, 1999; Stolarski et al., 2015), whereas a Past Negative orientation has been linked to dysregulation and maladaptive coping. Interoceptive awareness and temporal orientation can be understood as reciprocal processes: heightened bodily awareness may foster temporal balance, and temporal balance may support deeper interoceptive attunement. Together, these dimensions shape the regulatory environment through which conscious experience is embodied and stabilized.

Translationally, sleep and digestion offer accessible, experiential indicators of embodied regulation. In the present study, sleep and digestion were assessed using single-item self-rated scales, conceptualized as subjective markers of embodied functioning rather than diagnostic instruments. These measures were included to capture how regulatory processes manifest in lived experience, complementing trait-level assessments of interoceptive awareness and time perspective. While such indicators do not provide physiological measurement, they offer ecologically valid access to perceived autonomic regulation within everyday life. Sleep disturbance has been linked to diminished interoceptive awareness (Arora et al., 2021) and deviations in temporal orientation (Rönnlund et al., 2021; Herzog-Krzywoszanska et al., 2024). Gastrointestinal functioning, similarly, is sensitive to stress and autonomic imbalance, with functional GI disorders showing altered gut–brain interoceptive signaling (Karaivazoglou et al., 2024).

Recent empirical work further supports the connection between interoception and temporal processing using objective and performance-based measures. Studies employing heartbeat detection accuracy and cardiac monitoring paradigms have shown that interoceptive accuracy correlates with precision in time estimation, particularly at short intervals (Meissner and Wittmann, 2011; Uraguchi et al., 2022). Other research indicates that awareness of cardiac signals can attenuate emotion-induced distortions in perceived duration (Özoğlu and Thomaschke, 2020), while synchronization and tapping paradigms suggest that interoceptive sensitivity contributes to temporal control and precision (Teghil et al., 2020; Tomyta et al., 2023). Multimodal investigations further highlight dynamic brain-heart interactions during timing tasks, suggesting that bodily signals are integrated into neural timing processes (Richter and Ibáñez, 2021; Khoshnoud et al., 2024).

Contemporary models of interoception distinguish several related but conceptually distinct dimensions. Interoceptive accuracy refers to objective performance in detecting internal bodily signals, typically assessed using behavioral tasks such as heartbeat detection tasks. Interoceptive sensibility refers to individuals’ subjective beliefs or self-reported sensitivity to bodily signals, commonly measured through questionnaires. Interoceptive awareness has also been used to describe the metacognitive correspondence between subjective reports and objective performance (Garfinkel et al., 2015). Although these distinctions are theoretically meaningful, they represent complementary levels of analysis within the broader construct of interoception. The present study focuses specifically on subjective interoceptive awareness as assessed by the Multidimensional Assessment of Interoceptive Awareness (MAIA), which captures an individual’s capacity to notice, regulate, and interpret bodily sensations in everyday experience (Mehling et al., 2018).

Within this framework, much of the existing literature operationalizes interoception through objective accuracy measures and time through performance-based estimation tasks. The present study adopts a complementary approach, focusing on trait-level interoceptive awareness and stable temporal orientation as experiential dimensions of conscious regulation. Self-report measures such as the MAIA capture subjective aspects of bodily awareness that may not be accessible through laboratory accuracy tasks alone and are widely used to investigate experiential and regulatory dimensions of interoception (Mehling et al., 2018). By examining validated psychometric measures of both constructs, we extend existing findings from laboratory timing paradigms to the domain of embodied temporal perspective. This approach provides an important foundation for future studies integrating behavioral and physiological measures of interoception.

To our knowledge, no studies have examined these relationships at the trait level using validated psychometric measures of both constructs in relation to subjective autonomic indicators. Building on our previous theoretical review (Klamut and Weissenberger, 2023), the present study investigates whether interoceptive awareness and time perspective jointly shape embodied consciousness, operationalized through autonomic indicators such as sleep and digestion quality. These non-invasive measures provide a translational bridge between cognitive, emotional, and physiological dimensions of self-regulation. Sleep and digestion were selected as accessible indicators of autonomic regulation because both functions are closely linked to vagal activity and homeostatic stability. Rather than serving as direct measures of consciousness, they provide observable physiological outcomes through which embodied regulatory processes associated with conscious self-organization may be inferred, consistent with neurovisceral integration models linking autonomic regulation to adaptive cognitive and affective functioning (Thayer and Lane, 2009; Smith et al., 2017).

By situating these processes within a shared embodied and temporal framework, this work aims to contribute to methodologically grounded consciousness research. It highlights how consciousness can be accessed and investigated through its most universal entry points: the body and time.

## Materials and methods

### Participants

A total of 152 people took part in the survey, including 110 women (72.4%), 37 men (24.3%) and five people (3.3%) who described their gender as ‘other’. The age of participants ranged from 20 to 75 years (35.55 ± 9.19.)

A sensitivity power analysis conducted using G*Power 3.1 indicated that with *N* = 152, the study had 80% power to detect correlations of approximately *r* = 0.23 at *α* = 0.05 (two-tailed). This suggests the sample was adequately powered to detect small-to-medium effects within the range typically observed in individual-difference research.

### Measures

#### Zimbardo time perspective inventory–short form

Time perspective was assessed using the 18-item short version of the Zimbardo Time Perspective Inventory (ZTPI-SF), comprising five subscales: Past Negative, Past Positive, Present Hedonistic, Present Fatalistic, and Future. Each item was rated on a 5-point Likert scale ranging from 1 (very untrue) to 5 (very true). Subscale scores were calculated by averaging the items within each dimension. Cronbach’s alpha values indicated satisfactory internal consistency for most subscales: Past Negative (*α* = 0.77), Past Positive (*α* = 0.71), Present Hedonistic (*α* = 0.63), Present Fatalistic (= 0.52), and Future (= 0.73).

The five subscales represent distinct temporal orientations: Past Negative reflects a regretful or aversive view of one’s past; Past Positive captures a nostalgic, warm past orientation; Present Hedonistic reflects a focus on pleasure, novelty, and excitement; Present Fatalistic reflects a sense of helplessness or lack of control over the present; and Future reflects goal-orientation and planning.

To evaluate overall temporal balance, we computed the Deviation from Balanced Time Perspective (DBTP) index, which quantifies the Euclidean distance between an individual’s scores and a theoretically “optimal” profile. This profile—high Past Positive and Future, low Past Negative and Present Fatalistic, and moderate Present Hedonistic—was established in prior empirical research identifying adaptive time perspective patterns (Zimbardo and Boyd, 1999; Stolarski et al., 2011).

The Deviation from Balanced Time Perspective (DBTP) was computed using the formula by Stolarski et al. (2020):


$$\text{DBTP}=\sqrt{\begin{array}{l} {\left(\mathrm{oPN}-e\mathrm{PN}\right)}^{2}+{\left(\mathrm{oPP}-\mathrm{ePP}\right)}^{2}\\ +{\left(\mathrm{oPF}-e\mathrm{PF}\right)}^{2}+{\left(\mathrm{oPH}-e\mathrm{PH}\right)}^{2}+{\left(\text{oF}-\mathrm{eF}\right)}^{2} \end{array}}$$

Where o represents the optimal score and e the empirical score. Optimal values were: PN = 1.95, PP = 4.60, PF = 1.50, PH = 3.90, and *F* = 4.00. Higher DBTP scores indicate greater deviation from a balanced time perspective. Cronbach’s alpha was not applicable to the DBTP composite.

#### Multidimensional assessment of interoceptive awareness–version 2

Interoceptive awareness was measured using the MAIA-2, consisting of 37 items across eight subscales: Noticing (*α* = 0.79), Not-Distracting (*α* = 0.86), Not-Worrying (*α* = 0.78), Attention Regulation (*α* = 0.90), Emotional Awareness (*α* = 0.87), Self-Regulation (*α* = 0.87), Body Listening (*α* = 0.87), and Trusting (*α* = 0.95). Items were rated on a 6-point Likert scale from 0 (never) to 5 (always), and subscale scores were computed as item averages. Reliability estimates confirmed very good internal consistency across subscales.

#### Sleep and digestion quality

Participants self-reported their sleep and digestion quality using two single-item measures rated on a 5-point scale (1 = very poor, 5 = very good). These outcomes were treated as continuous variables in the analysis. As single-item variables, Cronbach’s alpha was not applicable.

### Procedure

Data collection took place between August and November 2023 using the online survey platform Tally.so. Participants were recruited through two main channels: social media outreach, including Facebook groups and posts related to psychology and well-being; and a scanable QR code poster distributed at the Mind and Life Europe Summer Research Institute (SRI), in Tuscany, Italy. The SRI was chosen as a recruitment site primarily for its availability as a large gathering of adults from diverse fields, including neuroscience, psychology, and contemplative studies. This provided access to an international, non-clinical sample well suited to the exploratory aims of the study.

Participants were directed to a website including a brief study introduction outlining the purpose and voluntary nature of the research. Participants were informed that the survey was anonymous and that they could withdraw at any point. Informed consent was implied by continuation of the survey.

Participants were first asked to provide their age, gender, and, optionally, their name and email address. Following this, they rated their sleep quality and digestion quality on 5-point Likert-type scales. They were also asked to report how often they typically get sick per year; however, this variable was not used in the present analysis.

Subsequently, participants completed two standardized self-report measures in a fixed order: the Zimbardo Time Perspective Inventory (ZTPI) and the Multidimensional Assessment of Interoceptive Awareness (MAIA-2). The total estimated time to complete the survey was approximately 15 min.

Participants were not formally screened for psychiatric, neurological conditions or medication use. Recruitment targeted a non clinical community sample, and participation was anonymous and voluntary; therefore, no clinical exclusion criteria was implemented. Future research may benefit from incorporating clinical screening procedures to further examine how such factors may influence the relationships observed in the present study.

### Study hypotheses

The present study was exploratory in nature, aiming to examine associations between interoceptive awareness, time perspective, and somatic functioning (sleep and digestion). Based on theoretical models linking interoception to autonomic regulation (Craig, 2009; Mehling et al., 2018) and research associating balanced time perspective with adaptive functioning (Zimbardo and Boyd, 2008; Stolarski et al., 2015), the following hypotheses were formulated.

*H1*: Higher interoceptive awareness is associated with a more balanced time perspective.

*H2*: A more balanced time perspective is associated with higher sleep quality.

*H3*: A more balanced time perspective is associated with higher digestion quality.

*H4*: Greater interoceptive awareness is associated with higher sleep quality.

*H5*: Greater interoceptive awareness is associated with higher digestion quality.

*H6*: Balanced time perspective mediates the association between interoceptive awareness and sleep quality.

*H7*: Past–negative time perspective mediates the association between interoceptive awareness and sleep quality.

### Data analysis

As this was a cross-sectional, correlational study, all hypotheses were tested at the level of associations rather than causal effects.

All statistical analyses were performed using IBM SPSS Statistics, Version 29.0 (Statistical Package of Social Science, IBM Corp.). Descriptive statistics were computed to summarize the quantitative variables.

Bivariate correlations were examined using Pearson’s r for continuous variables and Spearman’s rho for ordinal or non-normally distributed variables. Mediation analyses were conducted using the PROCESS macro for SPSS (version 5.0) (Hayes, 2022), specifically Model 4, with 5,000 bootstrap resamples and 95% bias-corrected confidence intervals to assess indirect effects. In addition, standard errors were estimated using the HC4 heteroskedasticity-consistent estimator (Cribari-Neto and Lima, 2009) to account for potential nonconstant error variance (Table 1).

**Table 1:** Descriptive statistics of quantitative variables for the whole sample.

Variables	*N*	*M*	SD	Mdn	Ske	K	IQR	α
Sleep quality	152	3.60	0.89	4.0	−0.45	0.54	1.00	N/A
Digestion quality	152	3.68	0.98	4.0	−0.59	0.05	1.00	N/A
1. Noticing	152	3.34	0.88	3.5	−0.77	1.23	1.25	0.79
2. Not-distracting	152	2.26	0.95	2.2	0.51	0.10	1.33	0.86
3. Not-worrying	152	2.50	0.86	2.6	−0.24	−0.50	1.40	0.78
4. Attention regulation	152	2.90	0.97	3.0	−0.64	0.39	1.29	0.90
5. Emotional awareness	152	3.57	1.05	3.8	−1.08	1.18	1.20	0.87
6. Self-regulation	152	2.94	1.11	3.1	−0.75	0.20	1.25	0.87
7. Body listening	152	2.64	1.17	2.7	−0.59	−0.13	1.50	0.87
8. Trusting	152	3.20	1.39	3.7	−0.74	−0.34	2.00	0.95
9. Past negative	152	2.99	1.00	3.0	0.05	−0.89	1.67	0.77
10. Past positive	152	3.43	0.83	3.7	−0.63	0.01	1.00	0.71
11. Present hedonistic	152	3.48	0.79	3.7	−0.46	0.10	1.00	0.63
12. Present fatalistic	152	2.23	0.68	2.0	0.43	−0.05	1.00	0.52
13. Future	152	3.33	0.74	3.5	−0.54	0.20	1.00	0.73
14. DBTP	152	2.44	1.00	2.4	0.36	−0.60	1.56	N/A

Ske – skewness, K – kurtosis, IQR -Interquartile Range.

## Results

Descriptive statistics for the main variables are presented in Table 2. The data included means (M), standard deviations (SD), medians (Mdn), skewness (Ske), kurtosis (K), interquartile range (IQR), and Cronbach’s alpha (*α*) where applicable. All variables showed acceptable levels of skewness and kurtosis, indicating no severe deviations from normality. Given the exploratory design and the number of associations examined, formal correction procedures for multiple comparisons were not applied. The findings should therefore be interpreted as hypothesis-generating rather than confirmatory.

**Table 2:** Correlation matrix of interoceptive dimensions and time perspectives (Addresses hypothesis H1).

	1	2	3	4	5	6	7	8	9	10	11	12	13	14
1. N	–													
2. ND	0.18^*^	–												
3. NW	−0.02	0.10	–											
4. AR	0.60^***^	0.25^**^	0.25^**^	–										
5. EA	0.70^***^	0.20^*^	−0.01	0.67^***^	–									
6. S-R	0.50^***^	0.29^***^	0.30^***^	0.75^***^	0.66^***^	–								
7. BL	0.65^***^	0.14	0.15	0.65^***^	0.74^***^	0.67^***^	–							
8. T	0.43^***^	0.26^**^	0.27^***^	0.66^***^	0.62^***^	0.71^***^	0.57^***^	–						
9. PN	−0.05	−0.34^***^	−0.39^***^	−0.21^**^	−0.08	−0.35^***^	−0.21^**^	−0.44^***^	–					
10. PP	0.22^**^	−0.09	0.02	0.15	0.25^**^	0.22^**^	0.25^**^	0.33^***^	−0.20^*^	–				
11. PH	0.27^***^	−0.09	−0.07	0.12	0.27^***^	0.20^*^	0.21^**^	0.24^**^	0.00	0.38^***^	–			
12. PF	0.24^**^	−0.01	−0.11	0.17^*^	0.28^***^	0.16^*^	0.28^***^	0.19^*^	−0.01	0.07	0.04	–		
13. F	0.15	0.29^***^	0.38^***^	0.34^***^	0.24^**^	0.46^***^	0.27^***^	0.46^***^	−0.56^***^	0.24^**^	0.04	−0.00	–	
14. DBTP	−0.11	−0.09	−0.29^***^	−0.21^*^	−0.16^*^	−0.36^***^	−0.22^**^	−0.43^***^	0.62^***^	−0.70^***^	−0.36^***^	0.21^**^	−0.58^***^	–

*N* = Noticing, ND = Not = Distracting, NW = Not = Worrying, AR = Attention Regulation, EA = Emotional Awareness, S = R = Self = Regulation, BL = Body Listening, T = Trusting, PN – Present Negative, PP – Present Positive, PH – Present Hedonistic, PF = Present Fatalistic, F – Future, DBTP = Deviation from Balanced Time Perspective. ^*^p < 0.05, ^**^p < 0.01, ^***^p < 0.001.

### Analysis of research hypotheses

#### Hypothesis 1

*H1*: Higher interoceptive awareness is associated with more balanced time perspective

To examine this hypothesis, Pearson correlation coefficients were computed between the eight subscales of the Multidimensional Assessment of Interoceptive Awareness (MAIA-2) and five time perspectives from the Zimbardo Time Perspective Inventory (ZTPI-S), along with the deviation from balanced time perspective (DBTP).

The analysis revealed statistically significant negative correlations between DBTP and six of the eight interoceptive subscales. Strongest associations were found for Trusting (*r* = −0.43, *p* < 0.001) and Self-Regulation (*r* = −0.36, *p* < 0.001), suggesting that greater interoceptive awareness in these domains is related to a more balanced time perspective.

Additional significant correlations were observed between specific interoception subscales and individual time perspective dimensions:

- Trusting was negatively associated with Past Negative (*r* = −0.44, *p* < 0.001) and positively with Past Positive (*r* = 0.33, *p* < 0.001), Present Hedonistic (*r* = 0.24, *p* < 0.01), and Future (*r* = 0.46, *p* < 0.001).
- Self-Regulation showed significant negative correlation with Past Negative (*r* = −0.35, *p* < 0.001) and positive correlations with Past Positive (*r* = 0.22, *p* < 0.01), Present Hedonistic (*r* = 0.20, *p* < 0.05), and Future (*r* = 0.46, *p* < 0.001).
- Emotional Awareness correlated positively with Past Positive (*r* = 0.25, *p* < 0.01), Present Hedonistic (*r* = 0.27, *p* < 0.001), and Present Fatalistic (*r* = 0.28, *p* < 0.001).
- Attention Regulation was positively correlated with Future (*r* = 0.34, *p* < 0.001) and negatively with Past Negative (*r* = −0.21, *p* < 0.01).
- Not-Worrying showed a negative correlation with Past Negative (*r* = −0.39, *p* < 0.001) and a positive correlation with Future (*r* = 0.38, *p* < 0.001).

These findings provide further support for Hypothesis 1, confirming that interoceptive awareness is meaningfully associated with time perspective profiles, particularly with a more adaptive, balanced configuration.

#### Hypothesis 2

*H2*: A more balanced time perspective is associated with higher sleep quality

To test this hypothesis, Spearman’s rank correlation coefficients were computed between sleep quality and time perspective dimensions, including DBTP. The results are presented in Table 3.

**Table 3:** Correlations of sleep quality with dimensions of interoception (*N* = 152; Addresses hypothesis H2).

Variable		PN	PP	PH	PF	F	DBTP
Sleep quality	rho	−0.40^**^	0.25^*^	0.07	0.09	0.25^*^	−0.27^*^

^*^p < 0.05, ^**^p < 0.01.

The analysis revealed that higher sleep quality was significantly associated with lower Past Negative orientation (r_s_ = −0.40, *p* < 0.001) and with higher scores on Past Positive (r_s_ = 0.25, *p* = 0.040) and Future orientations (r_s_ = 0.25, *p* = 0.030). Additionally, a significant negative correlation was found between sleep quality and DBTP (r_s_ = −0.27, *p* = 0.015), indicating that individuals with a more balanced time perspective tend to report better sleep quality (Table 4).

**Table 4:** Correlations of digestion quality with time perspectives (*N* = 152; Addresses hypothesis H3).

Variable		PN	PP	PH	PF	F	DBTP
Digestion quality	rho	−0.25^**^	0.08	0.11	0.11	0.19^*^	−0.13

^*^p < 0.05, ^**^p < 0.01.

Thus, Hypothesis 2 was supported.

#### Hypothesis 3

*H3*: A more balanced time perspective is associated with higher digestion quality

The analysis showed that digestion quality was significantly negatively correlated with Past Negative orientation (r_s_ = −0.25, *p* = 0.003), and positively associated with Future orientation (r_s_ = 0.19, *p* = 0.021). However, the correlation between digestion quality and DBTP was not statistically significant (r_s_ = −0.13, *p* = 0.115).

Therefore, Hypothesis 3 was not supported, as a more balanced time perspective (lower DBTP) was not significantly related to better digestion quality (Table 5).

**Table 5:** Correlations of sleep quality with dimensions of interoception (*N* = 152; Addresses hypothesis H4).

Variable		N	ND	NW	AR	EA	S-R	BL	T
Sleep quality	rho	0.10	0.19^*^	0.13	0.24^**^	0.16	0.29^**^	0.24^**^	0.38^**^

N = Noticing, ND = Not = Distracting, NW = Not = Worrying, AR = Attention Regulation, EA = Emotional Awareness, S = R = Self = Regulation, BL = Body Listening, T = Trusting ^*^p < 0.05, ^**^p < 0.01.

#### Hypothesis 4

*H4*: Greater interoceptive awareness is associated with higher sleep quality

The analysis revealed significant positive correlations between sleep quality and five subscales of interoceptive awareness. Specifically, sleep quality was positively related to Not-Distracting (r_s_ = 0.19, *p* = 0.023), Attention Regulation (r_s_ = 0.24, *p* = 0.004), Self-Regulation

(r_s_ = 0.29, *p* = 0.001), Body Listening (r_s_ = 0.24, p = 0.004), and Trusting (r_s_ = 0.38, *p* < 0.001). Among these, the strongest association was found for the Trusting dimension, suggesting that individuals who experience a greater sense of trust and connection with their body report better sleep quality. These results support Hypothesis 4 and indicate that specific aspects of interoceptive awareness may contribute to improved sleep (Table 6).

**Table 6:** Correlations of digestion quality with dimensions of interoception (*N* = 152; Addresses hypothesis H5).

Variable		*N*	ND	NW	AR	EA	S-R	BL	T
Digestion quality	rho	0.28^**^	0.23^**^	0.16^*^	0.28^**^	0.19^*^	0.26^**^	0.24^**^	0.37^**^

N = Noticing, ND = Not = Distracting, NW = Not = Worrying, AR = Attention Regulation, EA = Emotional Awareness, S = R = Self = Regulation, BL = Body Listening, T = Trusting ^*^p < 0.05, ^**^p < 0.01.

#### Hypothesis 5

*H5*: Greater interoceptive awareness is associated with higher digestion quality

The results indicated that digestion quality was significantly positively correlated with all eight subscales of interoceptive awareness. The strongest correlation was observed for Trusting (rs = 0.37, *p* < 0.001), followed closely by Noticing and Attention Regulation (both r_s_ = 0.28, *p* = 0.001). These findings suggest that individuals who report greater bodily awareness and emotional regulation also tend to perceive their digestion more positively.

Consequently, Hypothesis 5 was supported.

**Figure 1:**
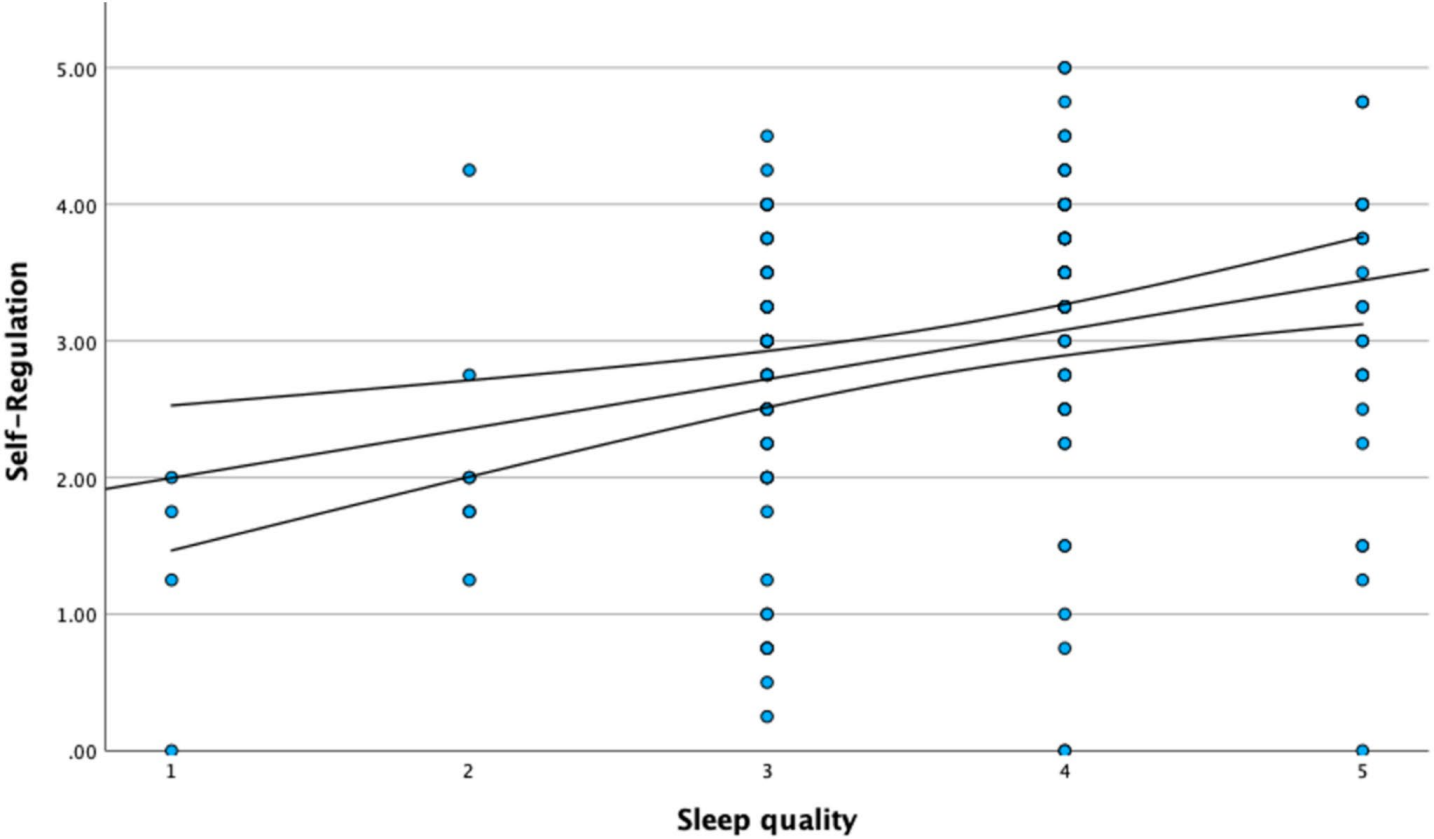
Relationship between self-regulation and sleep quality.

**Figure 2:**
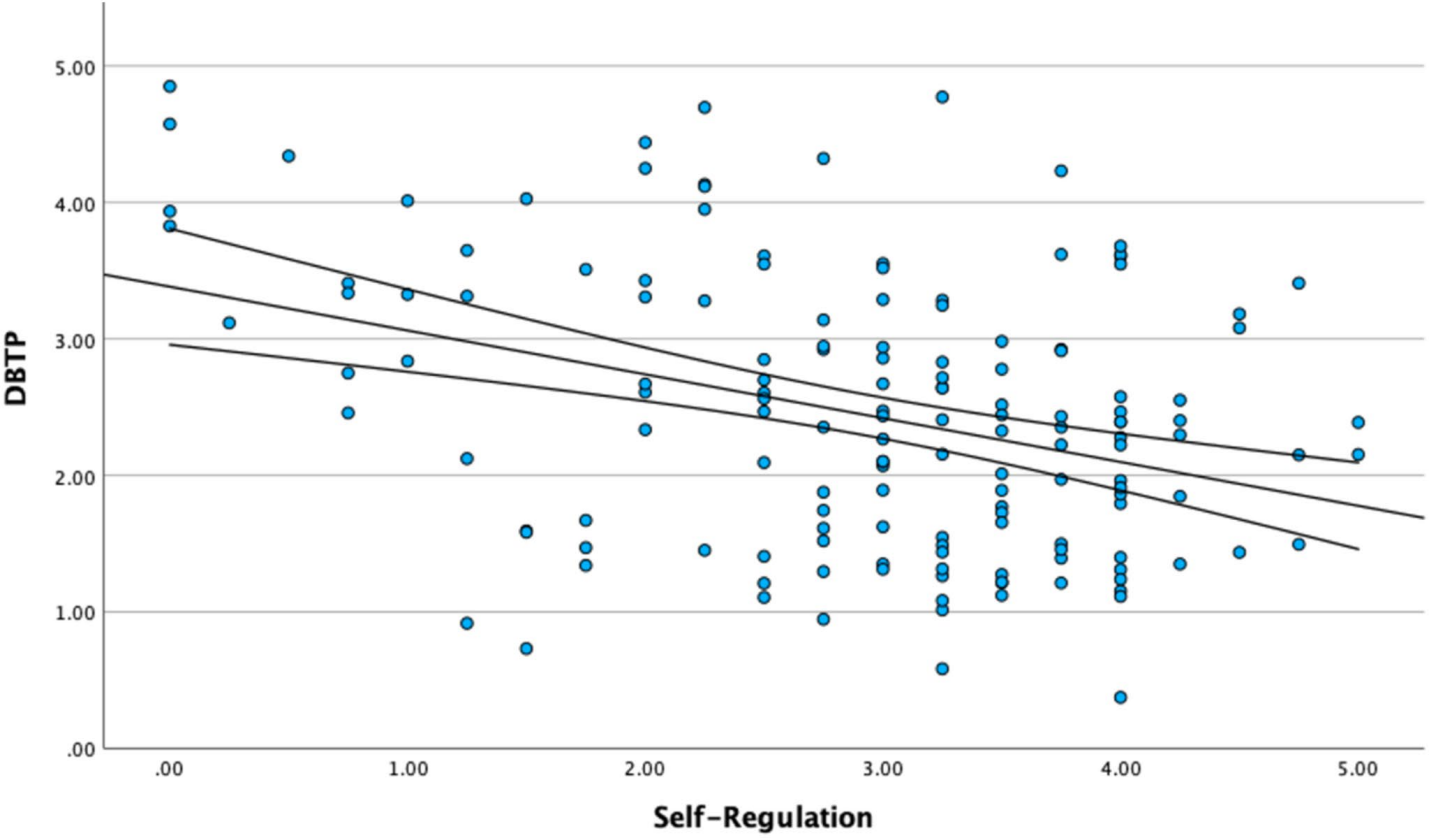
Relationship between self-regulation and deviation from balanced time perspective (DBTP).

**Figure 3:**
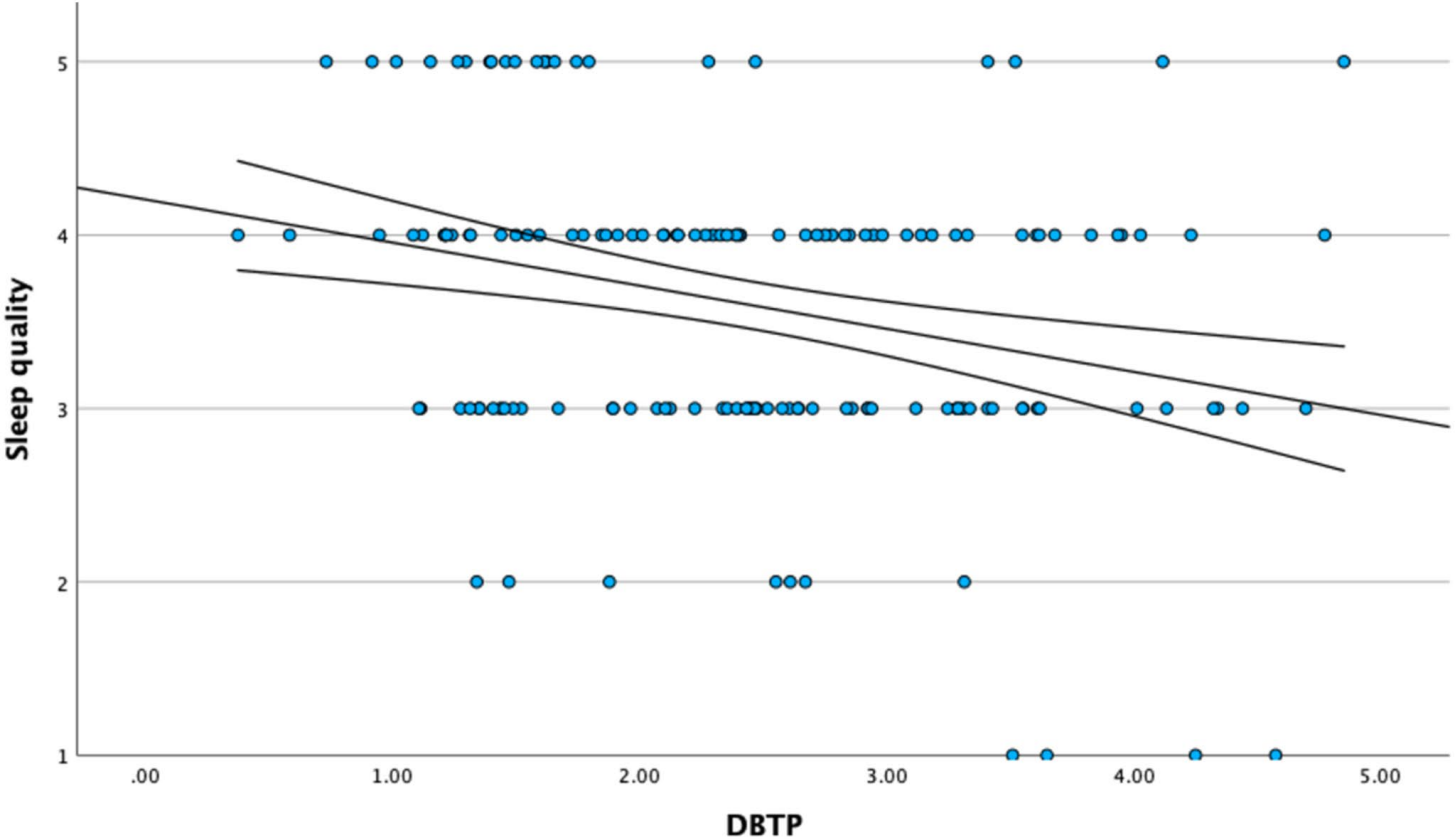
Relationship between deviation from balanced time perspective (DBTP) and sleep quality.

Figures 1–3 illustrate the primary bivariate associations underlying the proposed mediation model, with linear regression fits and 95% confidence intervals.

#### Hypothesis 6

*H6*: Balanced time perspective mediates the association of interoceptive awareness and sleep quality

To test whether balanced time perspective (DBTP) mediates the relationship between interoceptive awareness and sleep quality, mediation analyses were conducted using PROCESS (Model 4, version 5) in SPSS 29.0 (Table 7).

**Table 7:** Mediating role of DTP in relationship between self-regulation and sleep quality (Addresses hypothesis 6).

				95% CI		
Effect	Path	B	SE	LL	UL	*β*
Indirect effect	a × b	0.06	0.03	0.01	0.12	0.07
Components	a	−0.32	0.07	−0.46	−0.18	−0.36
	b	−0.18	0.09	−0.35	−0.01	−0.20
Direct effect	c’	0.17	0.08	0.02	0.33	0.22
Total effect	c	0.23	0.08	0.07	0.39	0.29

LL, Lower CI; UL, Upper CI.

The total effect of Self-Regulation on sleep quality was statistically significant, *β* = 0.29, 95% CI [0.07, 0.39]. When DBTP was introduced as a mediator, the direct effect remained significant, *β* = 0.22, 95% CI [0.02, 0.33]. The path from Self-Regulation to DBTP (path a) was significant, *β* = −0.36, 95% CI [−0.46, −0.18], and the path from DBTP to sleep quality (path b) was also significant, *β* = −0.20, 95% CI [−0.35, −0.01]. The indirect effect was *β* = 0.07, 95% CI [0.01, 0.15], supporting a partial mediation model.

**Figure 4:**
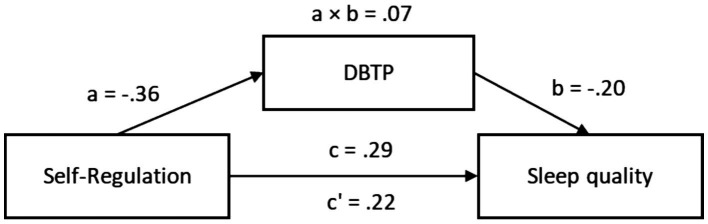
Indirect mediation model of self-regulation in sleep quality.

These results suggest that DBTP partially mediates the relationship between Self-Regulation and sleep quality. Individuals with higher self-regulatory interoceptive capacity are more likely to display a balanced time perspective, which in turn predicts better sleep outcomes. The partial mediation indicates that while DBTP accounts for part of the effect, Self-Regulation still has a direct influence on sleep quality (Figure 4; Table 8).

**Table 8:** Mediating role of DTP in relationship between attention regulation and sleep quality (*N* = 152; Addressing hypothesis 6).

				95% CI		
Effect	Path	B	SE	LL	UL	*β*
Indirect effect	a × b	0.05	0.03	0.01	0.11	0.05
Components	a	−0.21	0.09	−0.39	−0.03	−0.21
	b	−0.22	0.09	−0.39	−0.04	−0.24
Direct effect	c’	0.17	0.09	−0.01	0.35	0.19
Total effect	c	0.22	0.09	0.04	0.39	0.24

LL, Lower CI; UL, Upper CI.

**Figure 5:**
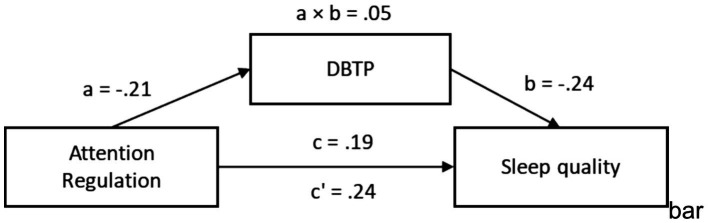
Indirect mediation model of attention regulation in sleep quality.

In the second model, the predictor was Attention Regulation. The total effect of Attention Regulation on sleep quality was significant, *β* = 0.24, 95% CI [0.04, 0.39]. When DBTP was introduced as a mediator, the direct effect of Attention Regulation on sleep quality was reduced and became nonsignificant, *β* = 0.19, 95% CI [−0.01, 0.35]. The path from Attention Regulation to DBTP (path a) was significant, *β* = −0.21, 95% CI [−0.39, −0.03], and the path from DBTP to sleep quality was also significant, *β* = −0.24, 95% CI [−0.39, −0.04]. The indirect effect was statistically significant, *β* = 0.05, 95% CI [0.01, 0.11], providing evidence for full mediation (Figure 5).

These findings confirm Hypothesis 6: time perspective, as operationalized by DBTP, significantly mediates the relationships between interoceptive awareness (specifically Self-regulation and Attention Regulation) and sleep quality.

#### Hypothesis 7

*H7*: Past-negative time perspective mediates the association of interoceptive awareness and digestion quality

A mediation analysis using the PROCESS macro (Model 4, version 5) tested whether Past-Negative time perspective mediates the relationship between Self-Regulation and digestion quality (Table 9).

**Table 9:** Mediating role of past negative time perspective in relationship between self-regulation and digestion quality (*N* = 152; Addressing hypothesis 7).

				95% CI		
Effect	Path	B	SE	LL	UL	*β*
Indirect effect	a × b	0.06	0.03	0.01	0.13	0.07
Components	a	−0.32	0.08	−0.48	−0.17	−0.35
	b	−0.20	0.09	−0.39	−0.02	−0.21
Direct effect	c’	0.19	0.09	0.00	0.37	0.21
Total effect	c	0.25	0.09	0.07	0.43	0.28

LL, Lower CI; UL, Upper CI.

**Figure 6:**
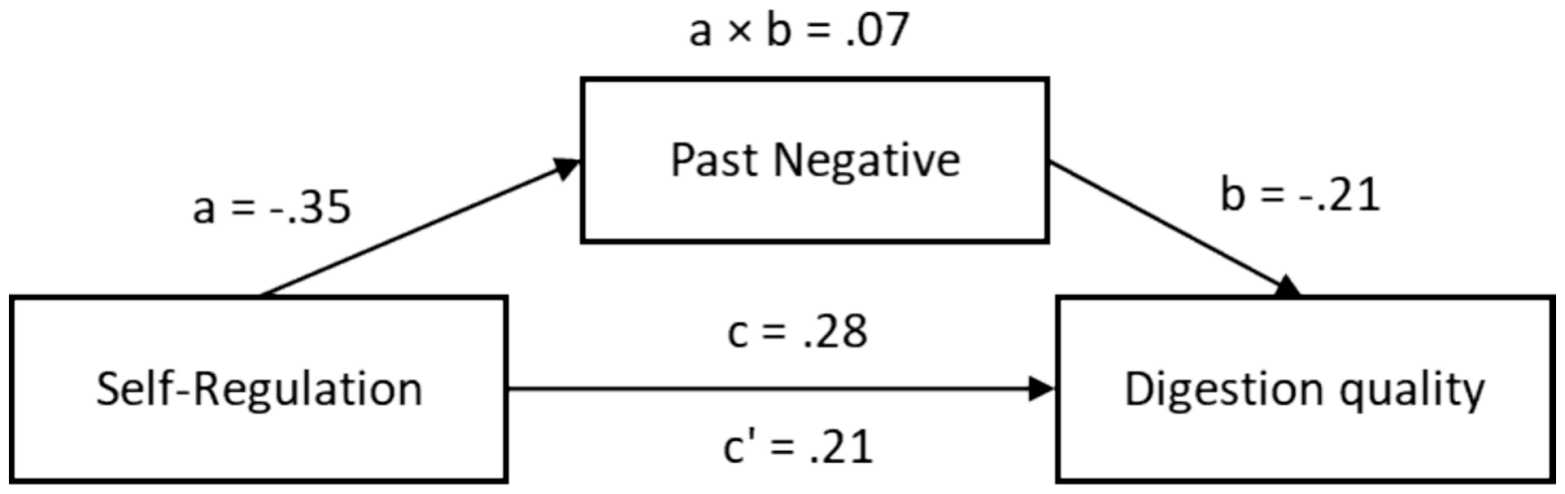
Indirect mediation model of self-regulation in digestion quality.

The total effect of Self-Regulation on digestion quality was significant, *β* = 0.28, 95% CI [0.07, 0.43]. When Past-Negative was included as a mediator, the direct effect remained significant, *β* = 0.21, 95% CI [0.00, 0.37]. The path from Self-Regulation to Past-Negative (path a) was significant, *β* = −0.35, 95% CI [−0.48, −0.17], as was the path from Past-Negative to digestion quality (path b), *β* = −0.21, 95% CI [−0.39, −0.02]. The indirect effect was statistically significant, *β* = 0.07, 95% CI [0.01, 0.15], suggesting partial mediation (Figure 6).

**Figure 7:**
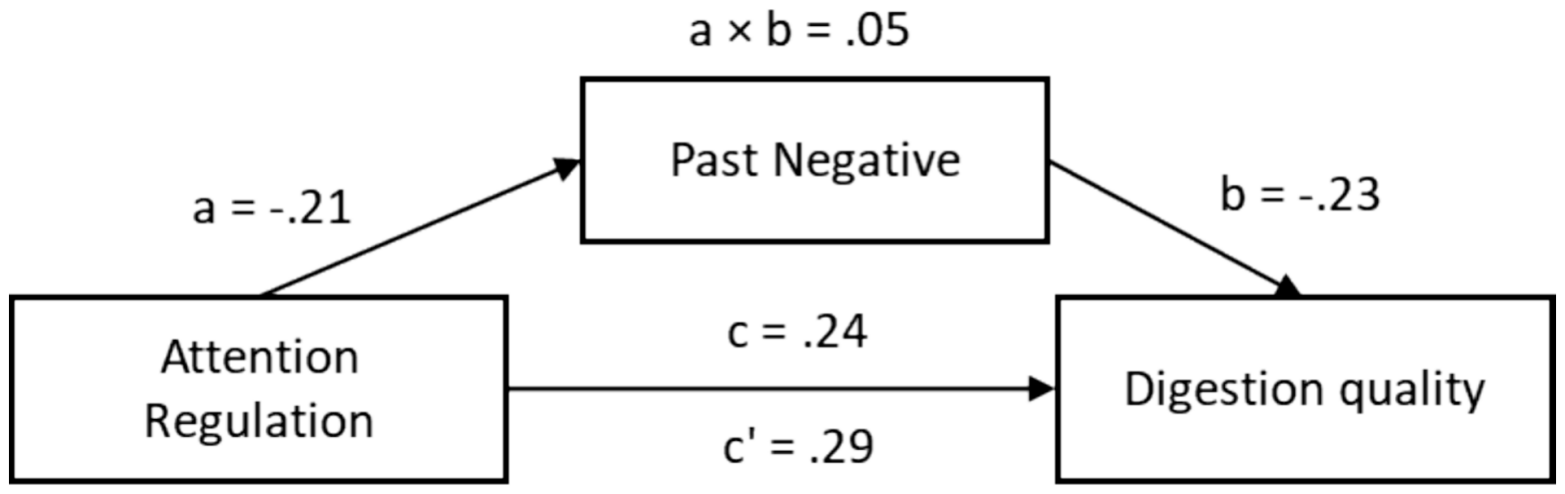
Indirect mediation model of attention regulation in digestion quality.

In a parallel analysis with Attention Regulation as the predictor, the total effect on digestion quality was also significant, *β* = 0.29, 95% CI [0.10, 0.48]. When Past-Negative was included as a mediator, the direct effect remained significant, *β* = 0.24, 95% CI [0.06, 0.42]. The path from Attention Regulation to Past-Negative (path a) was significant, *β* = −0.21, 95% CI [−0.42, −0.01], and the path from Past-Negative to digestion quality (path b) was also significant, *β* = −0.23, 95% CI [−0.40, −0.05]. The indirect effect was *β* = 0.05, 95% CI [0.00, 0.11], providing support for partial mediation (Figure 7; Table 10).

**Table 10:** Mediating role of past negative time perspective in relationship between attention regulation and digestion quality (*N* = 152; Addressing hypothesis 7).

				95% CI		
Effect	Path	B	SE	LL	UL	*β*
Indirect effect	a × b	0.05	0.03	0.00	0.11	0.05
Components	a	−0.22	0.10	−0.42	−0.01	−0.21
	b	−0.23	0.09	−0.40	−0.05	−0.23
Direct effect	c’	0.24	0.09	0.06	0.42	0.24
Total effect	c	0.29	0.10	0.10	0.48	0.29

LL, Lower CI; UL, Upper CI.

Together, these results indicate that both Self-Regulation and Attention Regulation are positively associated with digestion quality, and part of this relationship is explained by reduced past-negative time perspective.

## Discussion

This study contributes novel empirical evidence to the growing field of embodied consciousness research by examining how interoceptive awareness and time perspective jointly influence foundational indicators of autonomic regulation - specifically, self-rated sleep and digestion quality. Our findings suggest that the subjective capacity to detect and regulate internal bodily signals is associated with a more adaptive temporal orientation and, through that, with better somatic regulation. This supports the view that interoceptive awareness and temporal orientation function as interdependent regulatory systems, shaping both physiological homeostasis and the embodied structure of conscious experience.

### Interoceptive awareness and temporal orientation as anchors of consciousness

The observed association between key MAIA subscales (Self-Regulation, Trusting, Attention Regulation) and a balanced time perspective (lower DBTP) reinforces theoretical models that place bodily awareness and temporal orientation at the foundation of conscious self-regulation. Temporal balance reflects how individuals inhabit the present moment while maintaining coherent relations to past and future - a dynamic closely linked to the stability of conscious states.

This interpretation is consistent with Craig’s (2009) neuroanatomical model, which situates the anterior insula as a hub for interoceptive and emotional integration, and with Wittmann and Meissner (2022), who implicate the same region in subjective time perception and temporal continuity of the self. These mechanisms align with the neurovisceral integration model (Thayer and Lane, 2000; Smith et al., 2017), emphasizing the role of prefrontal, insular, and vagal pathways in maintaining adaptive regulatory loops.

More broadly, these results resonate with Seth and Tsakiris (2018), who propose that the self is grounded in interoceptive inference processes that underlie conscious experience. By linking interoception and temporal balance, our findings suggest that embodied temporal regulation may stabilize the sense of self in the present moment, providing a bridge between physiological regulation and subjective experience.

Additionally, among the MAIA subscales, Attention Regulation warrants particular consideration. This dimension indexes the capacity to sustain and flexibly direct attention toward bodily sensations. Its association with balanced time perspective is theoretically meaningful in light of established models of interval timing, in which attentional allocation governs the monitoring and accumulation of temporal information (Gibbon et al., 1984; Meck, 1996). While pacemaker–accumulator models conceptualize attention as a gating mechanism for short-interval timing (Gibbon et al., 1984; Meck, 1996; Meck and Benson, 2002), our findings suggest that trait-level attentional regulation may also relate to the broader organization of temporal experience.

From this perspective, attentional regulation may function as a shared mechanism linking interoceptive awareness and temporal orientation. The ability to sustain attention toward bodily signals could support more stable access to present-moment experience, which in turn may contribute to temporal balance. Importantly, this interpretation does not imply that attention alone generates conscious presence. Rather, attentional processes may facilitate access to interoceptive signals that contribute to the felt continuity of experience. In this way, attentional regulation may help connect short-interval timing mechanisms with the broader, embodied organization of temporal experience.

The mediation findings suggest temporal orientation represents a key psychological pathway linking interoceptive awareness to subjective autonomic functioning. From an embodied perspective, interoception provides present-moment physiological access, while time perspective situates these signals within cognitive-affective narratives of past and future.

These results align with theoretical models positing that embodied awareness and temporal cognition dynamically co-regulate conscious self-organization. Importantly, findings reflect statistical mediation within cross-sectional data, not causal transmission. Future longitudinal and experimental work can clarify these dynamics.

### Temporal orientation as a cognitive-affective filter

The finding that time perspective - particularly DBTP and Past Negative orientation - partially mediates the relationship between interoceptive awareness and somatic well-being suggests that temporal orientation functions as a cognitive-affective filter. Interoceptive signals are not merely perceived but appraised and situated in time. Individuals with more developed bodily awareness may be better equipped to interpret internal cues adaptively, thereby dampening stress reactivity and enhancing vagal recovery.

This mediation pathway extends prior work linking balanced time perspective to improved stress regulation (Stolarski et al., 2011) by identifying a new mechanism: temporal orientation as a bridge between bodily awareness and physiological regulation. In the context of consciousness, this illustrates how awareness of time and awareness of the body co-regulate the internal milieu, providing a stable scaffold for conscious presence (Northoff, 2014; Seth, 2021).

### Consciousness, autonomic regulation, and psychoneuroimmunology

The use of sleep and digestion as outcome variables situates these findings within the broader regulatory landscape of consciousness. These functions are tightly coupled with autonomic and affective states and are often disrupted across trauma, anxiety, and psychosomatic conditions (Chrousos and Gold, 1992; Harvey, 2002; Walker and Druss, 2016). Because they are directly modulated by vagal tone, they offer an accessible and biologically grounded entry point for studying how conscious states emerge and stabilize through bodily regulation.

Psychoneuroimmunological models provide additional explanatory power: chronic inflammation and early adversity alter long-term cognitive-emotional profiles through immune-to-brain signaling and neuroendocrine modulation (Ader et al., 1995; Bower and Kuhlman, 2023; Buric, 2025). Temporal biases, especially Past Negative orientation, may thus reflect embodied dysregulation, not merely learned cognitive patterns. Consciousness, in this framing, is not an isolated neural phenomenon but a systemic property emerging from dynamic interactions between neural, autonomic, and immune processes (Solms and Friston, 2018).

### Embodied time perspective as a bridge between lived experience and consciousness science

The concept of embodied time perspective offers a theoretical bridge linking interoception, temporal orientation, and conscious self-regulation. Interoceptive awareness anchors experience in the present moment, whereas temporal orientation situates that experience within a personal narrative of past and future. Together, they shape both the content and structure of conscious experience. This aligns with neurophenomenological and embodied accounts of consciousness that emphasize temporality as a constitutive dimension of experience (Northoff, 2014; Solms, 2021).

Our findings support the view that awareness of the body and awareness of time are reciprocal regulatory processes. Heightened interoceptive awareness may foster temporal balance, while temporal balance may enhance attunement to bodily signals. This bidirectional relationship offers a novel empirical paradigm for studying minimal consciousness through accessible and measurable physiological and psychological indicators.

### Future directions for consciousness research

This study is among the first to empirically demonstrate that time perspective statistically mediates the relationship between interoceptive awareness and subjective autonomic regulation. It contributes to an emerging methodological framework investigating consciousness through embodied and temporal dimensions, rather than through neural correlates alone.

Future research should employ longitudinal and experimental designs to clarify causal dynamics between interoception, temporal orientation, and conscious regulation. Integrating multimodal physiological markers (e.g., heart rate variability, cortisol, inflammatory cytokines) would allow for a more direct examination of brain–body–immune coupling in conscious states. Neuroimaging targeting insular, prefrontal, and vagal circuits may illuminate the neural mechanisms of embodied temporal integration. Finally, interventions combining interoceptive training (mindfulness), vagal stimulation, and time perspective therapy (Sword et al., 2015) could test whether modulating these processes alters conscious experience and physiological regulation.

## Limitations

Several limitations should be acknowledged. First, although the Zimbardo Time Perspective Inventory (ZTPI) offers a well-validated measure of temporal orientation, it primarily captures cognitive–affective aspects of time rather than embodied or phenomenological dimensions. As such, its scores may be influenced by dispositional affectivity, which was not assessed in this study. This raises the possibility of shared variance across interoception, time perspective, and somatic outcomes due to underlying mood states or affective traits. In addition, the exclusive reliance on self-report measures introduces the possibility of common method variance, which may inflate observed associations.

Second, the study’s cross-sectional and correlational design precludes causal inference. Alternative directions of influence or third-variable explanations cannot be excluded. While our mediation models were guided by existing evidence and theoretical models linking interoception, time perspective, and regulation, they should be interpreted as preliminary and exploratory: a statistical mediation within cross-sectional data rather than evidence of causal transmission. Longitudinal and interventional studies will be crucial to establishing causal pathways and testing reciprocal feedback mechanisms.

Third, the sample was predominantly female and relatively young, which may limit generalizability. Age and gender were not included as covariates in the mediation models, and future research should examine whether these relationships remain stable across demographic subgroups. Sex differences in interoceptive processing and autonomic regulation have been reported in prior research (Khalsa et al., 2018; Grabauskaitė et al., 2017), suggesting that more balanced samples may help clarify potential sex-specific effects. The absence of a comparison or control group further restricts the ability to explore demographic variability or assess differences across regulatory profiles.

Fourth, sleep and digestion were measured using single self-report items, which provide only coarse, subjective indices of these complex autonomic functions. Although such indicators offer accessible translational markers, future research would benefit from integrating multi-item scales and objective physiological indicators (such as polysomnography, heart rate variability, gut-brain biomarkers) to increase precision and ecological validity.

Finally, although the study was grounded in neurocognitive and embodied consciousness frameworks - including the neurovisceral integration model and insular involvement - our measures were limited to psychometric self-report instruments. No direct neural, physiological, or neurophenomenological data were collected. For this reason, the present findings should be regarded as a conceptual and empirical entry point, rather than a direct test of underlying mechanisms. Future studies incorporating multimodal physiological, neural, and experiential measures will be essential to advance this line of research and refine embodied paradigms for consciousness science. Moreover, given the number of statistical tests performed, future work should employ correction procedures for multiple comparisons to further strengthen inferential robustness.

## Conclusion

This study supports the conceptualization of interoceptive awareness and time perspective as dynamically interlinked dimensions of embodied consciousness, potentially embedded within shared neurovisceral pathways of regulation. By demonstrating that temporal orientation mediates the relationship between interoception and autonomic outcomes - specifically sleep and digestion quality - our findings offer a translational, embodied model in which cognitive-affective, physiological, and temporal processes converge to support adaptive self-regulation.

The observed mediation highlights temporal orientation as an important axis of embodied regulation. Rather than operating in isolation, interoception and time perspective appear to form a mutually reinforcing system: interoception provides access to present-moment bodily states, while temporal orientation offers a narrative and motivational framework for situating those signals within a broader temporal context. Together, they shape the regulatory environment through which conscious experience is organized and stabilized.

These results contribute to a growing literature emphasizing the interdependence of psychological, physiological, and experiential processes. Beyond their relevance to health and resilience, interoception and time perspective represent accessible, measurable entry points into the study of consciousness - linking subjective experience with embodied regulatory mechanisms.

As neuroscience moves toward integrative and embodied frameworks, these constructs may serve as key leverage points for developing new paradigms that bridge physiological regulation, temporal cognition, and conscious awareness. Future research integrating neural, physiological, and phenomenological measures will be necessary to clarify how these domains interact to shape the lived structure of conscious experience.

## Data Availability

The datasets presented in this article are not readily available because the dataset from this study contains some personal information (e.g., email addresses of participants who consented to be contacted with results) and therefore cannot be shared publicly in its original form. A de-identified version of the dataset is available on request from the corresponding author, to ensure participant confidentiality and compliance with data protection regulations. Requests to access the datasets should be directed to oklamut@gmail.com.
